# Estimating densities of large herbivores in tropical forests: Rigorous evaluation of a dung‐based method

**DOI:** 10.1002/ece3.4227

**Published:** 2018-06-27

**Authors:** Farshid S. Ahrestani, N. Samba Kumar, Srinivas Vaidyanathan, Lex Hiby, Devcharan Jathanna, K. Ullas Karanth

**Affiliations:** ^1^ Foundation for Ecological Research, Advocacy and Learning Tamil Nadu India; ^2^ India Program Wildlife Conservation Society Bengaluru India; ^3^ Conservation Research Ltd. Cambridge UK; ^4^ Centre for Wildlife Studies Bengaluru India; ^5^ Wildlife Conservation Society Global Conservation Program Bronx New York

**Keywords:** chital, decay rates, defecation rates, dung counts, DUNGSURV, elephant, gaur, muntjac, Nagarahole, sambar, wild pig

## Abstract

When sighting‐based surveys to estimate population densities of large herbivores in tropical dense forests are not practical or affordable, surveys that rely on animal dung are sometimes used. This study tested one such dung‐based method by deriving population densities from observed dung densities of six large herbivores (chital, elephant, gaur, muntjac, sambar, and wild pig) in two habitats, dry deciduous forests (DDF) and moist deciduous forests (MDF), within Nagarahole National Park, southern India. Using the program DUNGSURV, dung pile counts, decay rates estimated from field experiments, and defecation rates derived from literature were analyzed together by a model that allows for random events affecting dung decay. Densities of chital were the highest, followed by sambar. Wild pig densities were similar in the two habitats, sambar densities were higher in DDF, and densities of the other species were higher in MDF than in DDF. We compared DUNGSURV estimates with densities estimated using distance sampling in the same season. DUNGSURV estimates were substantially higher for all species in both habitats. These differences highlight the challenges that researchers face in computing unbiased estimates of dung decay rates and in relying on defecation rates from literature. Besides the elephant, this study is the first to rigorously test the efficacy of using a dung‐based approach to estimate densities of large herbivore species in Asia, and based on this evaluation, we provide specific recommendations to address issues that require careful consideration before observed dung densities are used to derive animal densities. Our results underline the need for an experimental study of a known population in a fenced reserve to validate the true potential of using dung‐based approaches to estimate population densities.

## 1 INTRODUCTION

Large herbivores are integral to terrestrial habitats across the Earth and as primary consumers are key to food webs and the functioning of ecosystems they inhabit (Gordon, Hester, & Festa‐Bianchet, 2004; Malhi et al., 2016). Populations of large herbivores across the earth have, however, collapsed, and many large herbivore species are threatened with large‐scale extirpations (Ripple et al., 2015). Assessing and prioritizing conservation action for these species depends on reliable information regarding their population sizes. The need to accurately estimate abundances of large herbivores has, therefore, never been greater.

Because of the difficulty of seeing animals in dense vegetation, deriving reliable estimates of large herbivore abundance in tropical forested landscapes has been problematic. The closed canopies of tropical forests make it difficult to assess populations from aerial surveys such as those used over open grasslands in East Africa and in temperate regions where forests lose their canopies in winter. Estimation techniques based on sighting animals (e.g., distance sampling using line transects, Buckland et al., 2001) often require large sampling efforts and substantial financial resources, which may not always be available (Karanth & Nichols, 2002). Researchers and managers have, therefore, attempted to estimate populations of large herbivores in tropical forests from their signs, instead of relying on direct observation of individuals (Kohn & Wayne, 1997; Putman, 1984). The most visible sign of a large herbivore in a forest is its dung, which it normally drops in a pile. These dung piles are relatively easy to find, identify, and count in forested habitats.

Counts of dung (pellet) piles were used for the first time to assess populations of big game in North America in the 1930s (Bennett, 1964; Bennett, English, & McCain, 1940). A subsequent seminal review by Neff (1968) then helped popularize dung counts as a tool to estimate populations. Although dung pile counts are used to asses populations primarily of large herbivores—mule deer *Odocoileus hemionus* in the United States (Collins & Urness, 1981), sika deer *Cervus nippon* in southern Scotland (Marques et al., 2001), white‐tailed deer *Odocoileus virginianus* in Mexico (Camargo‐Sanabria & Mandujano, 2011), roe deer *Capreolus capreolus* in Spain (Acevedo et al., 2010), kudu in South Africa (Ellis & Bernard, 2005), Asian elephant *Elephas maximus* (Hedges, Johnson, Ahlering, Tyson, & Eggert, 2013), and African elephants *Loxodonta africana* and *L. cyclotis* in Mozambique (Young, Ferreira, & van Aarde, 2009), Kenya (Vanleeuwe, 2009), and Gabon (Barnes et al., 1997)—the method has also been used to estimate populations of gorilla *Gorilla gorilla* (Todd, Kuehl, Cipolletta, & Walsh, 2008), hare *Lepus americanus* (Hodges & Mills, 2008), and badger *Meles meles* (Tuyttens et al., 2001).

Initial dung‐based models used to estimate population density assumed a *steady‐state* system, in which population density and defecation and dung decay rates remain constant long enough to result in a constant density of dung piles (McClanahan, 1986). For example, letting *E* represent elephant population density (elephants per square kilometer), *D* defecation rate (dung piles per elephant per day), and *α* the average number of days for which a dung pile is visible, the expected value of the dung pile density, *E*_*d*_ (elephant dung piles per square kilometer), equals *E* * *D* * *α*. A moment estimator for *E* is, therefore, *E*_*d*_ */Dα*.

In natural systems, *D* and *α* may vary with environmental conditions such as rainfall, temperature, food quality, and the impact of dung decomposition agents (Acevedo et al., 2010; Alves, da Silva, Soares, & Fonseca, 2013; Neff, 1968; Putman, 1984). To avoid the *steady‐state* assumption, alternative models have been proposed including models that treat dung decay iteratively (Plumptre & Harris, 1995); models that incorporate the role of rainfall influencing decay rates (Barnes, 2001; Theuerkauf & Gula, 2010); and models that allow for random events (e.g., rainfall, or a change in the abundance of decomposing agents) affecting dung decay (Hiby & Lovell, 1991; Laing, Buckland, Burn, Lambie, & Amphlett, 2003).

There is scarce literature on the use of dung counts to estimate population densities of large herbivores other than elephants in Asia (Hedges, 2012). To fill this knowledge gap, the Wildlife Conservation Society (WCS)‐India Program—as part of its long‐term predator–prey population monitoring project—planned this study to provide scientists, conservationists, and managers insight into using dung counts to estimate large herbivore abundances in tropical Asia. Thus, the goal of this study was to test the practicality and reliability of using dung pile counts to estimate population densities, which we did for six large herbivore species in two different habitats in a tropical forest in southern India. We used the Hiby and Lovell (1991) model, which does not assume a steady state and allows for random events affecting decay rates, to analyze dung counts and decay rates derived from the field along with defecation rates derived from literature. We then compared these estimates to density estimates derived from distance sampling—which relied on direct observations of species and not their dung—to assess the precision and accuracy of the dung‐based density estimates.

To our knowledge, this is the first study from Asia to test whether the use of dung counts is indeed a viable option to assess population densities of different large herbivore species in tropical forests where application of methods such as distance sampling and capture–recapture are impractical or cannot be implemented.

## 2 METHODS

**Figure 1:**
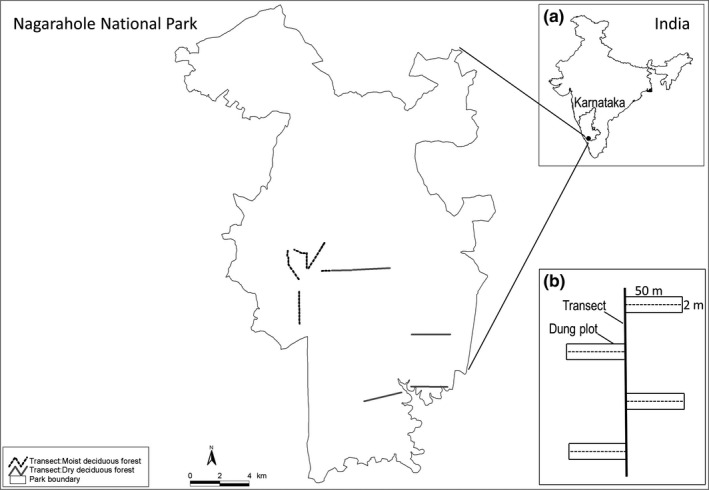
The location of the eight line transects in the dry deciduous forests (DDF; gray lines) and moist deciduous forests (MDF; black dotted line) found within Nagarahole National Park, southern India (insert a). The rectangular 100‐m^2^ plots in which dung piles were counted were located at 100‐m intervals perpendicular to and on alternate sides of the line transects (insert b)

We counted dung piles of six large herbivore species (elephant *Elephas maximus*, gaur *Bos gaurus*, sambar *Rusa unicolor*, chital *Axis axis*, muntjac *Muntiacus vaginalis*, and wild pig *Sus scrofa*) in plots located within two major habitats: dry deciduous (DDF) and moist deciduous (MDF) forests found within Nagarahole National Park, southern India (Figure 1). These two major habitats within our study area differed primarily in the mean annual rainfall they received: 1,500–2,100 mm in MDF, and 1,200–1,500 mm in DDF (Ramesh et al. 2011). Previous estimates from Nagarahole—WCS India's monitoring of large herbivores in Nagarahole is the longest‐running (1988–2017) animal‐monitoring project in South Asia—have shown that densities of large herbivores do differ between the two habitats.

The dung pile counts and decay rates were analyzed along with published defecation rates using the DUNGSURV model to estimate population densities of the large herbivore species (Hiby & Lovell, 1991). The DUNGSURV estimates of density were then compared to density estimates obtained from direct sighting‐based distance sampling using line‐transect surveys (Buckland et al. 2001). The dung counts were done immediately after the end of dung decay experiments and just before the distance sampling, ensuring that all parameters were estimated in the same season in the same year in the same area. Except for defecation rates, which were derived from the literature, all other parameters were estimated from field data.

### 2.1 Decay rate experiments

Prior to the dung counts, we conducted decay rate experiments from 14 November 1998 to 5 February 1999. At weekly intervals, we located an average of 25 (range: 3–46) fresh dung piles of each species. The samples were tagged and their GPS locations were recorded. A few days before the dung counts began, all the dung piles tagged as part of the decay rate experiment were revisited to record their final stage of decay. Dung piles were assigned to one of five states of decay, similar to those prescribed by (Barnes & Jensen, 1987) for elephants: A—fresh and wet with odor; B—completely dry with inner contents also completely dry; C—starting to decay and one or more pellets have decomposed; D—50% of the pile is decomposed, but still identifiable; and E—state of complete decay. At the end of the experiment, we were left with the percentages of each weekly sample of dung piles—all of which began in stage A (fresh)—that had reached other stages of decay.

### 2.2 Dung pile counts

In general, there are no problems in identifying and differentiating between a dung pile of an elephant, gaur, or a wild pig, nor confusing these with pellets dropped by any of the three deer species. Differentiating between the three deer species can, however, be tricky at times. Deer pellet size is related to body size; muntjac pellets are the smallest, chital are twice as large, and sambar pellets are the largest. In addition to size, pellets of the three deer species also differ in shape; both barking deer and chital pellets are narrow, long, and cylindrically shaped with a length:width ratio of 2:1, while a sambar pellet is distinctly different, i.e., it is rounded and is normally as long or longer than a chital pellet, but with a length:width ratio that rarely exceeds 1.5 (personal observations of the authors).

Body size remains an important factor as pellets of older, larger individuals are normally larger than pellets of younger, smaller individuals of all three deer species. On the rare occasion when a pile was difficult to identify as either sambar or chital—pellets of a young sambar can be sometimes very similar to pellets of an older chital—then the pile was not counted. Other challenges included cases when several dung piles of socially gregarious species like chital were found together, or when a pile was spread out. In general, very few piles were excluded as field personnel could rely on the experience of the WCS India program that had monitored large herbivores in Nagarahole for over a decade.

We specified that a dung pile must have a minimum of three pellets for deer, a minimum of one bolus for elephant, one dung pat for gaur, and one dropping for wild pig. We chose the standing crop (SC) approach over the accumulation rate (AR) approach for counting dung piles in sampling plots. Using the SC approach has been shown to increase precision of density estimates (Alves et al., 2013). The difference between the two approaches is that AR is measured by clearing sample plots of dung piles and then revisiting the plots to determine the rate of dung pile accumulation between two points in time, while SC is measured by counting dung piles in a plot without prior clearing or revisits (Acevedo et al., 2010; Putman, 1984).

Dung piles were counted (26 January–8 February 1999) once within 2 × 50 m plots spaced at 100 m intervals perpendicular to and on alternate sides of line transects (Figure 1b). These line transects were a part of the line‐transect system that WCS India had established for long‐term population monitoring of large herbivores using distance sampling. A total of 293 plots were sampled (162 in DDF and 131 in MDF) along 28.5 km (15.8 km in DDF and 12.7 km in MDF) of 11 line transects (five in DDF and six in MDF; see Supporting Information Table S1). All plots along a given transect were surveyed in <2 days, and given the long distances between transects, it was unlikely that either field activity or animal movement had any effect in biasing the dung pile counts along transects over the 14‐day survey period. To minimize the chance that dung piles were missed, a team of three observers searched the entire 50 m length of each rectangular sampling plot in unison. The observer in the middle recorded the counts and the two outer observers used 1‐m sticks to measure the width of the plot and moving aside any ground cover or leaf litter so that no dung pile remained hidden.

Each recorded pile was assigned to one of the four states of decay as defined in the decay rate experiment (except, obviously when a pile was found in a complete state of decay, i.e., stage E). The same field personnel who conducted the dung pile counts also located piles and monitored the decay process in the decay rate experiments, minimizing any observer bias among the two data collection methods.

### 2.3 Defecation rates

Defecation rates were derived from the literature (see Supporting Information Table S2). We used the mean defecation rate that had been reported for elephant, 18 piles/day (Hedges, 2012), wild pig, 4 piles/day (Ferretti, Storer, Coats, & Massei, 2015; Plhal, Kamler, & Homolka, 2014), and sambar, 21 piles/day (Rajapakshet, Padmalal, Kotagama, & Athulathmudali, 2013). For chital, two significantly different defecation rates have been reported, 12.6 (Rollins, Bryant, & Montandon, 1984) and 28 (Dinerstein & Dublin, 1982); as both rates were reported from captive animals that were fed forage that we were unsure matched what is found in southern Indian deciduous forests, we chose to use 28 piles/day as it would provide more meaningful density estimates. As we know of no defecation rates reported for the gaur we used the highest defecation rate (16 piles/day) reported for cattle (Phillips, 1993). For the Indian muntjac, we used the mean defecation rate of 8 piles/day reported for another muntjac species, the Reeve's muntjac (Chapman, 2004; see Table 1).

**Table 1:** Population densities of six large herbivore species in Nagarahole National Park, southern India, estimated by DUNGSURV using counts, defecation rates, and duration of decay of dung piles. Forest type: DDF = dry deciduous forest; MDF = moist deciduous forest

	Forest type	Defecation rate (piles/individual/day)	Density of dung piles/km^2^ (all decay stages)	Density of dung piles/km^2^ (only decay stage A)	Density estimates (individuals/km^2^) using all 4 stages of decay	Calibration factor *F*(*t*)	CV of *F*(*t*)	Density estimate (individuals/km^2^)
Chital	DDF	28	81,481	1358	43.36	925.12	0.06	46.11^2^
*Axis axis*	MDF	28	149,160	4427	89.42	659.41	0.07	128.15^2^
Asian elephant	DDF	18	4,753	123	4.09	795.96	0.05	2.79^3^
*Elephas maximus*	MDF	18	5,725	305	5.68	586.57	0.05	4.03^3^
Gaur	DDF	16	3,889	185	4.18	712.32	0.03	2.25^3^
*Bos gaurus*	MDF	16	6,565	687	11.36	344.20	0.07	7.31^3^
Muntjac	DDF	8	4,321	0	9.63	398.94	0.05	9.74^3^ a
*Muntiacus vaginalis*	MDF	8	6,870	153	17.58	366.65	0.02	16.86^3^ a
Wild pig	DDF	4	2,963	185	14.15	68.8	0.08	17.05^2^
*Sus scrofa*	MDF	4	2,061	153	16.32	126.24	0.05	16.32^4^
Sambar	DDF	21	42,778	864	31.43	1023.86	0.03	37.50^3^ a
*Rusa unicolor*	MDF	21	29,847	916	23.95	921.06	0.04	28.09^3^

Notes

a Dung piles from the earliest sample were found in all stages of decay (except Stage A) at the time of the survey. For such cases, we reported estimates using the first three^3^ stages of decay.

^2/3/4^ indicates the number of decay stages for which the densities were determined; that is, no dung piles from the earliest sample were found in these decay stages at the time of the survey.

### 2.4 The DUNGSURV model

The DUNGSURV model (Hiby & Lovell, 1991)—which estimates densities of large herbivores by combining dung pile counts, defecation rates, and decay rates—has two advantages over other modeling approaches: It requires less effort to determine decay rates; and critically, it does not assume a *steady state* in the system, which models such as McClanahan (1986) do. DUNGSURV depends on the simple fact that dung piles visible during a count were deposited in the time preceding the count, irrespective of whether the system is in a *steady state*. The density of dung piles seen in various decay stages during a survey, therefore, is related to the density of animals that existed within the survey region before the survey date.

Let *P*(*t*) represent the density of a large herbivore population within the study area at time *t*, and let *D*(*t*) represent the density of dung piles (e.g., number of dung piles per km^2^). If *P*(*t*), defecation rate *d* (dung piles deposited by an individual per day), and decay rate remain constant long enough for *D*(*t*) to reach equilibrium, then:

(1) D=Pdα,

where *α* is the mean time a pile remains visible.

Where a number of distinct morphological stages are recognized (Barnes & Jensen, 1987), the time for a dung pile to pass beyond a given stage can be used instead of the time it remains “visible”; in our case *D*(*t*), therefore, refers to the density of dung piles in all stages up to and including a given decay stage. Let *g*(*a*,* z*) represent the probability that a dung pile which was deposited at time *z* will still be visible at age *a*, or in other words has not decayed past a specific stage of decay at age *a* days. Let *d*(*z*) represent defecation rate at time *z*, and *t* the time when the actual dung counts are carried out, then

(2) $$D\left(t\right)=\int_{t-T}^{t}d\left(z\right)g\left(t-z,z\right)P\left(z\right)dz,$$

where *t* – *z* is *a*, and *T* is any time interval large enough such that the probability a dung pile remains visible over interval *T* is negligible, that is, *g*(*T*,* z*) ≈ 0. We know that *d*(*z*)*P*(*z*)*dz* is the amount of dung produced per unit area over the time increment *dz*, and *g*(*t* – *z*,* z*) is the fraction that remains at the time of the survey, *t*. Therefore, the integral in equation (2) represents the sum of the number of dung piles that remain in different stages at the time of the survey.

If we assume that *P*(*z*) is constant at *P* over the period *t* – *T* to *t*, then *P*(*z*) can be taken out of the integral. If we let *F*(*t*) represent the remaining integral, $\int_{t-T}^{t}d\left(z\right)g\left(t-z,z\right)dz$, then equation (2) can be rewritten as

(3) D(t)=PF(t).

*F*(*t*) is the calibration factor relating dung density at time *t* to population density, and *P* is estimated as *D*(*t*)/*F*(*t*) by the program DUNGSURV. As *P* is our main parameter of interest, we need to remove it from the integral, which we do by multiplying and dividing the right‐hand side of equation (2) by *F*(*t*):

(4) $$D\left(t\right)=\frac{\int_{t-T}^{t}d\left(z\right)g\left(t-z,z\right)P\left(z\right)}{\int_{t-T}^{t}d\left(z\right)g\left(t-z,z\right)}F\left(t\right).$$

We see that *D*(*t*)/*F*(*t*) estimates a weighted average of the population density in the period preceding the survey, with weighting function *d*(*z*)*g*(*t* – *z*,* z*). If the calculation of *F*(*t*) is based on a late decay stage (e.g., stage D), then the population density estimate will be the average density over a longer period leading up to the survey date. In contrast, if *F*(*t*) is based on a recent decay stage (e.g., stage B), then the estimate will be weighted toward a shorter period of time.

The (DOS‐based) program DUNGSURV (http://conservationresearch.org.uk/Home/AdditionalPrograms.html) calculates densities using the formulae given above. The precision of estimates by DUNGSURV is increased if all dung pile samples being monitored from the first week of the decay rate experiment reach a stage of complete decay (stage E) by the end of the experiment. For the majority of species in this study, however, dung piles from week one had reached only decay stage C by the end of the experiment.

### 2.5 Distance (line transect) sampling by direct observation

In forests where sighting‐based surveys for wildlife are possible, comparing estimates based on dung surveys with estimates based on distance sampling surveys—if conducted at the same time—could prove useful (Buckland et al., 2001). Therefore, following the dung counts, we used the same line transects—along which we counted dung piles—to estimate densities of the six study species using distance sampling (Buckland et al., 2001, 2007). The 11 permanent transects we used—which were part of a long‐term monitoring system—ensured adequate geographical coverage as well as proportional representation of the two major habitat types available in the study area. Transects varied in length from 0.8 to 3.5 km and the total length of all 11 transects was 28.4 km. Each transect was sampled 26 times during 3–20 May 1999, which amounted to a total sampling effort of 738.4 km.

Transect lines were sampled on foot in the early mornings and late evenings when the probability of sighting the study species was highest. Data were collected by two trained observers on each transect walk who collected data every time they sighted a cluster (i.e., ≥1 individual) of any of the study species. The three field‐based parameters measured at each sighting of a cluster of a study species were (a) cluster size; (b) radial sighting distance from the midpoint of a cluster to the position of the observer; and (c) compass bearing along the transect line and to the cluster sighted. Data gathered from (b) and (c) were then used to compute the perpendicular distance from the animal cluster to the transect line. For further details of the distance sampling field protocols used, please refer to Karanth, Thomas, and Kumar (2002).

For each of the species, the set of perpendicular distance measurements enabled estimating a detection function and mean probability of detecting each species in the area sampled. By combining the detection function estimates with the number of clusters (*n*) and their respective sizes (*C*_s_), we were able to estimate the abundance parameters of interest: encounter rate (*n/l*;* l *= length of sampled transect line), species cluster density (*C*_D_), and species density (S_D_). These parameters of interest were estimated using the DISTANCE software, the most widely used analytical tool for distance sampling of animals (Thomas et al., 2010).

DISTANCE helps select the detection probability model based on the lowest Akaike information criteria (AIC) value, as well as on other model selection criteria like the *p*‐value of Kolmogorov–Smirnov test and the precision of parameter estimates (Buckland et al., 2001; Thomas et al., 2010). This final analytical step, however, was executed only after an initial exploratory analysis that assessed potential assumption violations and identified optimal strip widths to exclude outliers, which in turn helped choose the best‐fit detection probability function for a species. We included each habitat type as a stratum in DISTANCE and obtained stratum‐specific estimates of all distance sampling‐related parameters for each species (see Supporting Information Table S4). For further details of the analyses, please refer to Thomas and Karanth (2002).

Dung survey‐based density estimates are a weighted average of population density over a time interval preceding the survey, whereas distance sampling‐based estimates are an instantaneous snap shot of population density prevalent at the time of the survey. Hence, we compared density estimates from both methods for only those species not known to migrate in or out of the survey region during the overall sampling duration (time interval that covered both dung and distance sampling; see Table 3). On this basis, we compared DUNGSURV and distance sampling‐based estimates of elephant in MDF and sambar, chital and wild pig in both DDF and MDF (Table 3). We did not compare estimates of elephant in DDF as elephants were known to leave the DDF area and move to the periphery of a reservoir in the time period between when the dung and distance‐based surveys were conducted; muntjac in either habitat as a high percentage of the earliest sample in the muntjac's decay rate experiment had not decayed past stage C; and gaur in either habitat as a high percentage of the earliest sample in the gaur's decay rate experiment had not decayed past stage D.

## 3 RESULTS

Dung piles of chital, followed by sambar, were the most abundant in both forest types (Table 1; Supporting Information Table S3). The counts of wild pig dung piles were similar in the two habitats, counts of dung piles of sambar were higher in DDF than in MDF, whereas counts of the dung piles of all other species were higher in MDF than in DDF. Dung piles of all three deer species (muntjac, chital, and sambar) were found mostly in decay stage B (i.e., completely dry with inner contents also completely dry), dung piles for the two largest species (gaur and elephant) were found mostly in decay stage D (i.e., 50% of a pile was decomposed, but still identifiable), and dung piles of wild pig were found mostly in decay stage C (i.e., the pile had started to decay as one or more pellets were found decomposed; see Supporting Information Table S3).

**Figure 2:**
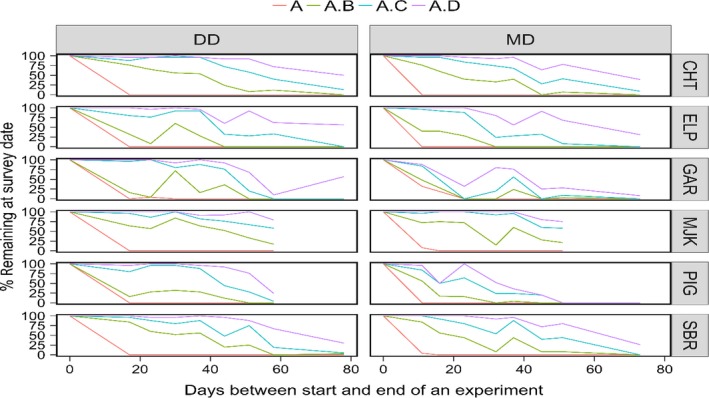
The percentage (%) of dung piles that remained in different stages of decay from the consecutive trials of the decay rate experiments of the different species at the time the final dung count survey was conducted. The species are CHT—chital; ELP—elephant; GAR—gaur; MJK—muntjac; PIG—wild pig; SBR—sambar. The legend labels are A—piles in stages A; A.B—piles in stages A and B; A.C—piles in stages A–C; and A.D—piles found in stages A–D. The two habitats are DD—dry deciduous forest; MD—moist deciduous forest

To increase the precision of our estimates, we hoped that all dung piles being monitored from week one of the decay rate experiment would reach a state of complete decay. In this study, however, all dung piles of only wild pig in MDF from week one had reached a complete state of decay by the time of the dung count survey (Figure 2). The estimate of wild pig in MDF, therefore, is the only density estimate that used data from all four stages of decay (refer to wild pig population density of 16.32^4^/km^2^ in MDF, Table 1; the four in the superscript indicates that data from all four stages were used). The population densities for muntjac are probably overestimated because a large proportion of its week one sample had not decayed past stage C (see muntjac MJK plots in Figure 2).

Defecation rates of chital (28 piles/day) and sambar (21 piles/day) were the highest among the study species, and the densities of these species estimated by DUNGSURV were the highest too (Table 1). Similar to the patterns in dung counts, estimated wild pig populations densities were similar in both habitats, sambar densities were higher in DDF than in MDF, and the other species were found at higher densities in MDF than in DDF (Table 1).

The distance sampling results revealed that encounter rate of chital was substantially higher in MDF than in DDF; the encounter rates for sambar and muntjac were higher in DDF than in MDF; and the encounter rates for elephant, gaur, and wild pig were similar in both DDF and MDF (Table 2). Truncation distances—the perpendicular distance from transect lines used by data analysts to discard larger distances from distance sampling datasets to facilitate robust estimation of detection functions (see Buckland et al., 2001 for a description of the truncation distance)—for gaur and pig were the widest (100 m), while the truncation distance of the muntjac data set was the narrowest (54 m; see Supporting Information Table S4). The encounter rate, the number of clusters detected, and the expected cluster size were the highest for chital in both habitats, which mirrored the fact that chital density estimates were highest among all species in both habitats (Table 2). This was not the pattern, however, for the other species. For example, although the number of clusters detected, the encounter rate, and expected cluster size were all higher for gaur than for sambar in MDF, a much smaller effective strip width—the perpendicular distance from transect lines at which number of detections beyond the strip width will equal the number of detections missed within the strip width (see Buckland et al., 2001 for a definition of effective strip width)—for sambar resulted in higher densities of sambar than gaur in MDF (Table 2).

**Table 2:** Data used and parameters estimated by DISTANCE of six large herbivore species in Nagarahole Tiger Reserve, southern India, using data collected by distance‐based line‐transect sampling. Forest type: DDF = dry deciduous forest; MDF = moist deciduous forest

Species	Forest type	No. of clusters detected	No. of clusters used	Encounter rate (clusters/km)	Effective strip width (m)	Cluster density (clusters/km^2^)	Expected cluster size	Density estimate (individuals/km^2^)	*SE* of density estimate
Chital	DDF	131	119	0.29	23.3	6.22	2.48	14.43	3.23
*Axis axis*	MDF	304	237	0.70	45.3	7.72	6.34	48.95	7.99
Asian elephant	DDF	52	45	0.11	41.3	1.33	2.62	3.48	1.06
*Elephas maximus*	MDF	38	34	0.1	46.1	1.08	2.03	2.20	0.78
Gaur	DDF	68	64	0.16	31.6	2.47	2.89	7.14	1.90
*Bos gaurus*	MDF	68	58	0.17	41.5	2.06	5.86	12.10	3.62
Muntjac	DDF	61	57	0.14	25.5	2.73	1.1	2.97	0.75
*Muntiacus vaginalus*	MDF	27	25	0.07	17.4	2.13	1.0	2.13	0.84
Wild pig	DDF	35	35	0.09	31.1	1.37	2.57	3.53	1.22
*Sus scrofa*	MDF	44	40	0.11	32.6	1.81	1.63	2.95	0.93
Sambar	DDF	103	101	0.25	27.6	2.17	1.57	3.42	1.19
*Cervus unicolor*	MDF	44	35	0.1	23.8	4.46	1.75	7.81	1.53

The one trend shared among the estimates by DISTANCE and DUNGSURV was that except for wild pig, for which densities were similar in the two habitats, all other species were at higher densities in MDF than in DDF (Tables 1 and 2). Besides this common trend, the densities estimated by DISTANCE were, without exception, substantially lower than the DUNGSURV estimates for each species in both habitats (Table 3).

**Table 3:** Comparison of animal densities (individuals/km^2^) estimated by DUNGSURV and Distance in the dry deciduous forests (DDF) and moist deciduous forests (MDF) of Nagarahole National Park, southern India

	Forest type	DUNGSURV	DISTANCE (*SE*)*
Chital	DDF	46.11^2^	14.43 (3.23)
*Axis axis*	MDF	128.15^2^	48.95 (7.99)
Asian elephant *Elephas maximus*	MDF	4.03^3^	2.20 (0.78)
Wild pig	DDF	17.05^2^	3.53 (1.22)
*Sus scrofa*	MDF	16.32^4^	2.95 (0.93)
Sambar	DDF	37.50^3^	7.81 (1.53)
*Rusa unicolor*	MDF	28.09^3^	3.42 (1.19)

Note

^2/3/4^ indicates the number of decay stages for which the densities were determined; that is, no dung piles from the earliest sample were found in these decay stages at the time of the survey.

## 4 DISCUSSION

Despite using a theoretically robust model that improved upon earlier models by not assuming a *steady‐state* system, we nevertheless ended with dung‐based density estimates that were substantially larger than distance sampling estimates. We also invested substantial effort and resources to collect field data on dung densities and conduct decay rate experiments to estimate population densities for the six large herbivore species. However, quantifying the level of uncertainty of *F*(*t*), the calibration factor in the DUNGSURV model that relates dung pile densities to population densities, seems almost impossible given its dependence on temporal and spatial variation in defecation and decay rates. The coefficients of variation (CVs) of *F*(*t*) are almost certainly underestimated because they assume the piles included in the sample at time *z* are a pure random sample from all piles deposited at time *z* throughout the entire study area. In practice, though, we were probably capturing the variance in decay rate at a local scale, which may have been different from the mean decay rate for the entire study area. This can be a major issue as temporal and, particularly, spatial variations in decay rates of dung piles in any area are generally extremely high (Hedges, 2012).

Although not expressed explicitly in the *g*(*t* – *z*,* z*) function, dung decay is affected by factors that vary spatially as well as temporally. Three different habitat conditions (closed canopy, open canopy, and bare soil) were recognized within each of the two major habitat types (MDF and DDF). These may have affected decay rates differently and explain why for some species, such as gaur in DDF (see Figure 2), dung samples that we began monitoring later in the experiment, had reached a more advanced stage of decay than dung piles that we began monitoring earlier in the experiment. It is possible that if the distribution of fresh dung piles over the three habitat conditions was identical in each sample, we may have had decay rate curves that declined monotonically with age for all species in both habitats.

To check the robustness of the DUNGSURV model, we ran a simulation for elephant in MDF using a known elephant density, the oft‐used defecation rate of 18 piles per elephant/day, and decay rates similar to those observed in our decay experiments (see Supporting Information Appendix S1). Given the simulated dung count and decay rate data, the elephant density estimated by the Hiby and Lovell (1991) model was very similar to the elephant density used in the simulation (see Supporting Information Appendix S1), demonstrating that DUNGSURV can estimate densities accurately, provided reliable defecation rates, decay rates, and dung densities can be obtained.

Another reason for the differences in density estimates between the two methods could be the use of defecation rates from literature, which were at best from other sites and captive animals, and at worst were of other species. The food available to the study species in the wild most likely differed from the diet fed to the captive animals used to estimate the published defecation rates we used (except for elephant, for which defecation rates were measured in the field). It is also highly likely that behavioral changes brought about by captivity influenced the published defecation rates. Furthermore, we had no choice but to rely on defecation rates of surrogate species; we used the published defecation rate of cattle for the gaur, and the one published defecation rate of Reeve's muntjac—from a single study on captive animals housed in England—for the Indian muntjac (see Supporting Information Table S2).

Even if we assume that piles of chital remain fresh for two consecutive days in early February when we counted dung piles—the realistic number of days that piles would remain fresh at the onset of the summer season, which is typically February to April—the distance sampling‐based density estimate of 14.4 chital/km^2^ in DDF cannot be equated to the 1,358 fresh (stage A) chital dung piles/km^2^ that were counted during our survey. To equate the count of 1,358 fresh chital dung piles to a density estimate of 14.4 would require a defecation rate of 47 piles/day, which we very much doubt was the case for chital. One reason for the discrepancy between the chital estimates could be that the distance sampling estimates were downward biased. Distance sampling estimates of density can be biased, either upward or downward, when assumptions of line‐transect sampling are not fully satisfied (Buckland et al., 2001; Thomas & Karanth, 2002). Although we followed field and analytic protocols correctly (Karanth et al., 2002), it is plausible that the fundamental assumptions of line‐transect sampling—such as animals directly on the line are always detected and that animals are detected at their initial location prior to any movement in response to the observer—were violated and biased the density estimates. On the other hand, though we were confident that we never missed a dung pile within a plot during our counts, it is still possible that our dung counts were inflated. For example, a single dung pile deposited by, say, chital or a sambar while walking might have been counted as two or more piles by the surveyors. Other reasons for inflated counts could be the plot shape, which had a relatively high edge:area ratio (rectangular versus circular), and the use of multiple observers.

To validate the dung approach and iron out any issues that could bias accurate counts of dung piles, it would be useful to conduct a study on a known population of a large herbivore species within a fenced wildlife reserve. Testing of different plot sizes, shapes, and placements would be possible, and regular exhaustive searches for fresh dung would provide information on defecation rates, how best to define a dung pile based on average pellet numbers deposited, along with large samples for decay rate experiments. Importantly, resulting dung‐based animal density estimates could be compared with a known animal density unaffected by various uncontrollable factors in the field.

Even if levels of uncertainty cannot be established, researchers may sometimes be faced with the need to estimate population density in situations where sighting‐based surveys are either impossible, not affordable, practical, or feasible. If dung surveys are used in such situations, we recommend conducting the dung counts toward the end of a period of relatively constant population density, uniform dung decay, and during a period of good visibility for dung piles (e.g., end of the dry season). To increase the accuracy of dung counts, we recommend using the same team of observers to count dung piles in all the plots. In the tropics, we recommend conducting the dung decay experiment over ~15 weeks to ensure complete decay of dung piles from the earliest samples before the commencement of dung count surveys. The decay rate experiments should include fresh dung samples found randomly over as wide an area as possible and frequently enough to capture the variance of decay rates in the study area. Any pile washed away by rain before the dung count survey date should be recorded as fully decayed at that time (i.e., beyond stage D).

In conclusion, this study clearly shows that invoking the impossibility of conducting sighting‐based surveys does not justify the naïve application of dung‐based approaches to estimate population abundance. At the very least, careful consideration of the various factors that could bias estimates of defecation rates, decay rates, dung densities, and, ultimately, animal densities is required. These are important as the reasons that dictate obtaining density estimates of threatened species require that the estimates we obtain are reliable if they are to usefully inform conservation action.

## CONFLICT OF INTEREST

None declared.

## AUTHOR CONTRIBUTIONS

NSK, UK, LH, and SV designed the study. NSK and SV were instrumental in data collection. LH developed software and model. FSA, DJ, SV, NSK, and LH analyzed the data. FSA wrote most of the manuscript.
