# Supplementary material for: Estimating densities of large herbivores in tropical forests: Rigorous evaluation of a dung‐based method
Source: Ecol Evol. 2018 Jun 27;8(15):7312–22. doi: 10.1002/ece3.4227 (PMC6106164; doi:10.1002/ece3.4227)
Supplement: Supplementary file 1 [file ECE3-8-7312-s001.docx]

**Supplementary material - 1**

**Table S1** The lengths of line transects used in distance sampling-based surveys, and along which dung piles were counted in plots (perpendicular to transects) for estimating population densities of large herbivore species in the dry deciduous forests (DDF) and moist deciduous forests (MDF) in Nagarahole National Park, southern India.

| Transect | Distance  (km) | Number  of dung plots | Habitat  type |
| --- | --- | --- | --- |
| 1 | 3.5 | 36 | DDF |
| 2 | 2.0 | 20 | DDF |
| 3 | 3.5 | 36 | DDF |
| 4 | 3.4 | 35 | DDF |
| 5 | 3.4 | 35 | DDF |
| Subtotal (DDF) | 15.8 | 162 |  |
| 6 | 1.4 | 14 | MDF |
| 7 | 1.5 | 15 | MDF |
| 8 | 3.2 | 33 | MDF |
| 9 | 3.1 | 32 | MDF |
| 10 | 2.7 | 28 | MDF |
| 11 | 0.8 | 9 | MDF |
| Subtotal (MDF) | 12.7 | 131 |  |
| Total (DDF+MDF) | 28.5 | 293 |  |

**Table S2** Daily defection rates of the study species derived from literature.

| Species | No. of dung piles /day/individual | Reference |
| --- | --- | --- |
| chital *Axis axis* | 12.6 | [Rollins, Bryant and Montandon (1984](#_ENREF_10)) |
|  | 28.0 | [Dinerstein and Dublin (1982](#_ENREF_3)) |
| Asian elephant *Elephas maximus*^¶^ | 18 | [Hedges (2012](#_ENREF_6)) |
| gaur *Bos gaurus** | 15.8 | [Aland, Lidfors and Ekesbo (2002](#_ENREF_1)) – cattle *Bos Taurus* |
|  | 10-16 | [Phillips (1993](#_ENREF_7)) – cattle *Bos Taurus* |
|  | 7.5 (summer)  5.4 (winter) | [Eycott *et al.* (2013](#_ENREF_4)) – European bison *Bison bonasus* |
| muntjac *Muntiacus vaginalis* | 7-8.2 | [Chapman (2004](#_ENREF_2)) – Reeves’s muntjac *Muntiacus reevisi* |
| wild pig *Sus scrofa* | 3.8-4.3 | [Ferretti *et al.* (2015](#_ENREF_5)) |
|  | 4.3 | [Plhal, Kamler and Homolka (2014](#_ENREF_8)) |
| sambar *Rusa unicolor* | 21 | [Rajapakshet *et al.* (2013](#_ENREF_9)) |

* There exists no data on defecation rates for the gaur. Therefore, we report rates of closely related species.

^¶^ Hedges (2012) established the defecation rate of 18 for the Asian elephant after reviewing 16 studies that had reported defecation rates for elephants

**References for Table S2**

Aland, A., Lidfors, L. & Ekesbo, I. (2002) Diurnal distribution of dairy cow defecation and urination. *Applied Animal Behaviour Science,* **78,** 43-54.

Chapman, N.G. (2004) Faecal pellets of Reeves’ muntjac, Muntiacus reevesi: defecation rate, decomposition period, size and weight. *European Journal of Wildlife Research,* **50,** 141-145.

Dinerstein, E. & Dublin, H.T. (1982) Daily defecation rate of captive axis deer. *The Journal of Wildlife Management,* **46,** 833-835.

Eycott, A., Daleszczyk, K., Drese, J., Cantero, A.S., Pèbre, J. & Gladys, S. (2013) Defecation rate in captive European bison, Bison bonasus. *Acta Theriologica,* **58,** 387-390.

Ferretti, F., Storer, K., Coats, J. & Massei, G. (2015) Temporal and spatial patterns of defecation in wild boar. *Wildlife Society Bulletin,* **39,** 65-69.

Hedges, S. (2012) Estimating elephant population density and abundance from dung pile density: Theoretical concepts. *Monitoring elephant populations and assessing threats: a manual for researchers, managers and conservationists* (ed. S. Hedges), pp. 61-111. Universities Press, Hyderabad, India.

Phillips, C.J.C. (1993) *Cattle Behaviour*. Farming Press Books, Ipswich.

Plhal, R., Kamler, J. & Homolka, M. (2014) Faecal pellet group counting as a promising method of wild boar population density estimation. *Acta Theriologica,* **59,** 561-569.

Rajapakshet, I., Padmalal, U., Kotagama, S. & Athulathmudali, N. (2013) Estimating the abundance of sambar deer (Cervus unicolor unicolor) at the Horton Plains National Park of Sri Lanka LANKA. *Proceedings of International Forestry and Environment Symposium*.

Rollins, D., Bryant, F.C. & Montandon, R. (1984) Fecal pH and defecation rates of eight ruminants fed known diets. *The Journal of Wildlife Management,* **48,** 807-813.

**Table S3** Counts of dung piles of six large herbivore species in Nagarahole National Park, southern India, classified into four stages of decay. Stages of decay: A - Fresh & wet with odour; B - Completely dry with inner contents also completely dry; C - Starting to decay and one or more pellets have decomposed; and D - 50% of the pile is decomposed, but still identifiable. Forest type: DDF = Dry Deciduous Forest; MDF = Moist Deciduous Forest.

| Species | Forest type | Total | | Stage A | | Stage B | | Stage C | | Stage D | |
| --- | --- | --- | --- | --- | --- | --- | --- | --- | --- | --- | --- |
|  |  | *raw* | */km^2^* | *raw* | */km^2^* | *raw* | */km^2^* | *raw* | */km^2^* | *raw* | */km^2^* |
| chital  *Axis axis* | DDF | 1320 | 81481 | 22 | 1358 | 669 | 41296 | 505 | 31173 | 124 | 7654 |
|  | MDF | 1954 | 149160 | 58 | 4427 | 1049 | 80076 | 663 | 50611 | 184 | 14046 |
| Asian elephant  *Elephas maximus* | DDF | 77 | 4753 | 2 | 123 | 11 | 679 | 23 | 1420 | 41 | 2531 |
|  | MDF | 75 | 5725 | 4 | 305 | 9 | 687 | 18 | 1374 | 44 | 3359 |
| gaur  *Bos gaurus* | DDF | 63 | 3889 | 3 | 185 | 6 | 370 | 17 | 1049 | 37 | 2284 |
|  | MDF | 86 | 6565 | 9 | 687 | 5 | 382 | 19 | 1450 | 53 | 4046 |
| muntjac  *Muntiacus vaginalis* | DDF | 70 | 4321 | 0 | 0 | 32 | 1975 | 31 | 1914 | 7 | 432 |
|  | MDF | 90 | 6870 | 2 | 153 | 65 | 4962 | 14 | 1069 | 9 | 687 |
| wild pig  *Sus scrofa* | DDF | 48 | 2963 | 3 | 185 | 16 | 988 | 23 | 1420 | 6 | 370 |
|  | MDF | 27 | 2061 | 2 | 153 | 6 | 458 | 11 | 840 | 8 | 611 |
| sambar  *Rusa unicolor* | DDF | 693 | 42778 | 14 | 864 | 353 | 21790 | 255 | 15741 | 71 | 4383 |
|  | MDF | 391 | 29847 | 12 | 916 | 192 | 14656 | 135 | 10305 | 52 | 3969 |

*Note*: To derive dung density estimates per km^2^, we divided the DDF *raw* dung counts by 0.0162 and the MDF counts by 0.0131 as we had sampled a total area of 16200 m^2^ (162 100m^2^ plots) in DDF and a total area of 13100 m^2^ (131 100m^2^ plots) in MDF, respectively. For example, the 1320 dung piles of CHT counted in DDF translated to a density of 81,481 piles/km^2^.

**Table S4** Data related to the detection functions that best fit to DISTANCE sampling data for the different study species

| Species | Forest  type | Truncation width (m) | Selected  model | Adjustment terms | Criteria used to select model |
| --- | --- | --- | --- | --- | --- |
| chital | DDF | 80 | Uniform cosine | 5 | AIC, visual assessment of fit |
| *Axis axis* | MDF | 80 | Uniform cosine | 2 | AIC, CV(p), visual assessment of fit |
| Asian elephant | DDF | 85 | Half Normal cosine | 1 | K-S test, visual assessment of fit |
| *Elephas maximus* | MDF | 85 | Half Normal cosine | 1 | K-S test, visual assessment of fit |
| gaur | DDF | 100 | Half Normal cosine | 1 | AIC, CV(p), visual assessment of fit |
| *Bos gaurus* | MDF | 100 | Half Normal cosine | 1 | AIC, CV(p), visual assessment of fit |
| muntjac | DDF | 54 | Half Normal cosine | 1 | AIC, CV(p), K-S test |
| *Muntiacus vaginalus* | MDF | 54 | Half Normal cosine | 1 | AIC, CV(p), K-S test |
| wild pig | DDF | 100 | Half Normal cosine | 3 | CV(p), visual assessment of fit |
| *Sus scrofa* | MDF | 100 | Half Normal cosine | 3 | CV(p), visual assessment of fit* |
| sambar | DDF | 70 | Uniform cosine | 2 | visual assessment of fit |
| *Cervus unicolor* | MDF | 70 | Uniform cosine | 3 | visual assessment of fit |

- CV(p): Coefficient of variation of detection probability (0-W); W = truncation width
- AIC not used as the model with the min. AIC model (Hazard Rate) fit data poorly close to the transect line.
- K-S test = Kolmogorov-Smrinov test; a higher p-value indicated a better fit
