# Supplementary material for: Estimating densities of large herbivores in tropical forests: Rigorous evaluation of a dung‐based method
Source: Ecol Evol. 2018 Jun 27;8(15):7312–22. doi: 10.1002/ece3.4227 (PMC6106164; doi:10.1002/ece3.4227)
Supplement: Supplementary file 2 [file ECE3-8-7312-s002.docx]

**Supplementary material – 2**

**Simulation to test DUNGSURV**

To validate the DUNGSURV model and software with data typical of decay rates and dung counts observed in the field, we simulated data similar to the elephant dung counts and decay experiment results from the moist deciduous forest (MDF) environment.

Assuming an animal density of 4 elephants/km^2^, and a defecation rate of 18 dung piles/elephant/day, a steady-state dung pile density in each of the 4 decay stages was generated using the following percentages of dung piles decaying/day from one stage to the next: 60% from stage A to stage B; 10% from stage B to stage C; 5% from stage C to stage D; and 2.5% from stage D to fully decayed. The resulting cumulative dung pile densities in stage A, stages A and B, stages A to C and stages A to D were 120, 840, 2280 and 5150 dung piles/km^2^; as compared to 305, 992, 2366, 5725 for the elephant data we found in the field (See Supplementary Material, Table S3).

Based on the transition probabilities from decay stage to decay stage that we found in our study, we simulated a decay rate experiment using the same intervals of times as in the actual field experiment. For the simulation, each experiment started with 25 fresh dung piles and Table S4 shows the number of piles from each experiment in each decay stage by the date of the end of the experiment (26/01/1999). Figure S1, which illustrates the simulated decay experiment results, shows that there were still some dung piles from the earliest experiment trial that remained in stage D at the end of the experiment. Therefore, the integral—representing density of dung piles at the time of the survey—for all stages combined underestimates the duration of the dung piles and thus overestimates animal density (* in Table S5). As no piles remained in stage B when using the simulated data (Table S4) we could use data for stages A to B, which provides an elephant density of 4.122/km^2^ (Table S5), and as no piles remained in stage C for the observed data (Table S4) we could use data for stages A to C, which provides an elephant density of 3.919/km^2^ (Table S5) as compared to the 4 elephants/km^2^ used to drive the simulation.


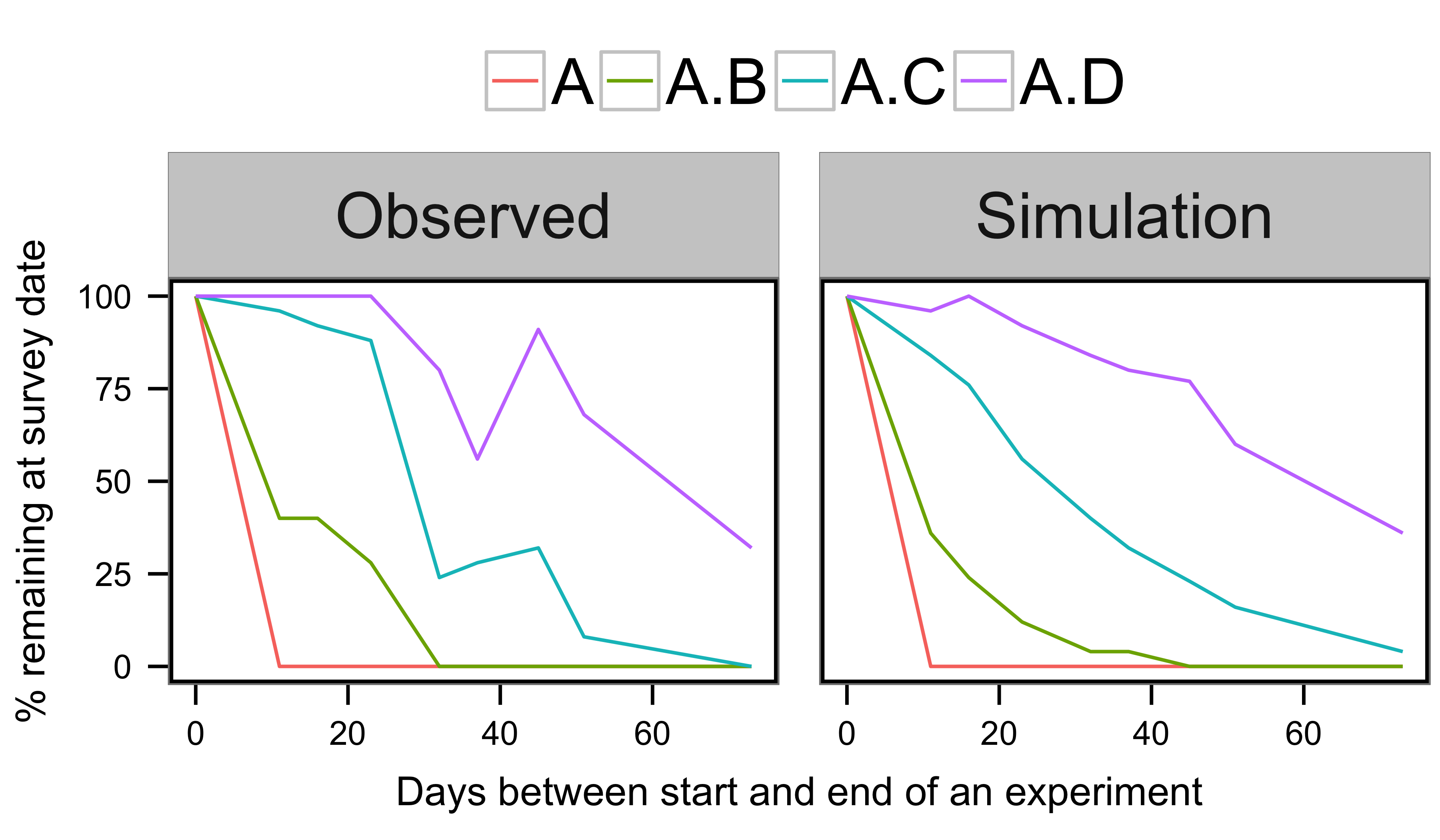


**Figure S1** The percentage (%) of dung piles from a series of decay rate samples—simulated for elephant dung piles in moist deciduous forest—that had reached different stages of decay at the time of the final survey. The decay rates were simulated based on the rates found in the study. The legend labels were: A – piles in stages A; A.B – piles in stages A and B; A.C – piles in stages A-C; and A.D – piles found in stages A-D.

**Table S5** Number of dung piles found in different stages of decay on date of final inspection (26/01/1999) from a series of decay rate experiments. The *Simulated data* were generated based on transition probabilities derived from our study’s decay experiment, and the *Observed Data* were derived using the same transition probabilities found in the field.

| Experiment start date | Observed data | | | | Simulated data | | | | |
| --- | --- | --- | --- | --- | --- | --- | --- | --- | --- |
|  | A | B | C | D | A | B | C | D |  |
| 14/11/1998 | 0 | 0 | 0 | 8 | 0 | 0 | 1 | 8 |  |
| 06/12/1998 | 0 | 0 | 2 | 15 | 0 | 0 | 4 | 11 |  |
| 12/12/1998 | 0 | 0 | 7 | 13 | 0 | 0 | 5 | 12 |  |
| 20/12/1998 | 0 | 0 | 7 | 7 | 0 | 1 | 7 | 12 |  |
| 25/12/1998 | 0 | 0 | 6 | 14 | 0 | 1 | 9 | 11 |  |
| 03/01/1999 | 0 | 7 | 15 | 3 | 0 | 3 | 11 | 9 |  |
| 10/01/1999 | 0 | 10 | 13 | 2 | 0 | 6 | 13 | 6 |  |
| 15/01/1999 | 0 | 10 | 14 | 1 | 0 | 9 | 12 | 3 |  |

**Table S6** shows the DUNGSURV output resulting from the input file of simulated decay experiment results and the simulated dung densities.

| Dung piles  in: | Dung pile density  (/km) | Elephant density^§^ (/km) | Calibration factor (CV) ^§^ | Elephant density^¶^ (/km) | Calibration factor (CV) ^¶^ |
| --- | --- | --- | --- | --- | --- |
| Stages A to D | 5150 | 5.166* | 997 (0.035) | 5.227* | 985 (0.038) |
| Stages A to C | 2280 | 3.919 | 582 (0.045) | 4.191* | 544 (0.063) |
| Stage A and B | 840 | 3.498 | 240 (0.091) | 4.122 | 204 (0.099) |
| Stage A | 120 | 1.212 | 99 (---) | 1.212 | 99 (---) |

^§^ Densities estimated using the *observed data* from Table S4

^¶^ Densities estimated using the *simulated data* from Table S4

* Densities estimated are biased as some dung piles from the first experiment still remained in decay stage C or D
